# Horticultural Therapy Program for People with Mental Illness: A Mixed-Method Evaluation

**DOI:** 10.3390/ijerph17030711

**Published:** 2020-01-22

**Authors:** Andrew M.H. Siu, Michael Kam, Ide Mok

**Affiliations:** 1 Department of Rehabilitation Sciences, The Hong Kong Polytechnic University, Hunghom, Hong Kong SAR, China; 2 New Life Psychiatric Rehabilitation Association, Tuen Mun, Hong Kong SAR, China; michaelkam@nlpra.org.hk (M.K.); ide@nlpra.org.hk (I.M.)

**Keywords:** horticulture, mental health, mixed methodology, psychiatric disability

## Abstract

Background. Horticultural therapy (HT) has long been used in the rehabilitation of people with mental illness, but many HT programs are not standardized, and there have been few evaluation studies. Aims. This study evaluated the process and outcomes of a standardized horticultural program using a mixed methodology, i.e., systematic integration (“mixing”) of quantitative and qualitative data within a study. Methods. Participants who have mental illnesses were assigned to a treatment (HT) and a comparison group (*n* = 41 for each group). The process and outcomes of the program, including stress and anxiety, engagement and participation, affect changes, mental well-being, and social exchange, were obtained using self-completed questionnaires, observational ratings of participants during the group, as well as through a focus group. Results. The study results supported the proposal HT is effective in increasing mental well-being, engagement, and the sense of meaningfulness and accomplishment of participants. Many participants reported a reduction in stress and anxiety in the focus group, but positive changes in affect were not fully observed during the group process or captured by quantitative measures. The participants also did not report increases in the social exchange over the HT sessions. Conclusion. The evidence supports that HT is effective in increasing mental well-being, engagement in meaningful activities, but did not result in significant affect changes during therapy, or increase social exchanges among people with mental illness.

## 1. Introduction

Horticultural therapy (HT) “is the engagement of a client in horticulture activities facilitated by a trained therapist to achieve specific and documented treatment goals” [1]. Considering the functional capacities of participants, therapists should set up relevant, practical, and measurable goals in one or more emotional, social, physical, and intellectual domains. While the end products of HT are expected to bring about a sense of accomplishment, there are several common features that facilitate the engagement and therapy process of HT: (1) Participants are asked to take care of at least one kind of plant during therapy; (2) participants are guided to experience the growth process of the plant(s); and (3) therapists should make use of the multi-sensory characteristics of plants during the program [2].

Horticulture has been long used as a therapeutic activity for people with mental illnesses, such as schizophrenia and depression [3,4]. People’s interactions with plants, through goal-orientated horticultural activities in the form of active gardening, as well as the passive appreciation of nature, could be therapeutic to people with mental disorders in many ways [5,6]. First, horticulture could have emotional benefits, such as reducing stress, reducing psychiatric symptoms, stabilizing mood, and increasing the sense of tranquility, spirituality, and enjoyment [1,7,8,9,10]. Second, it could help people to reduce fatigue and restore attention and cognitive ability [11,12,13]. Third, it could increase self-efficacy, self-esteem, and quality of life [5,14,15]. Fourth, horticultural therapy could provide a forum for developing group cohesiveness and a sense of belonging [16,17]. Fifth, it could help people to engage in purposeful activities and develop a sense of accomplishment and productivity [14,18]. Last, people with disabilities could develop sustainable vocational skills and productivity in horticulture [19]. Many existing reviews focused on the general benefits of HT for the general public and occasionally for people with health conditions [20,21]. Few studies have systematically investigated the outcomes and process of horticultural therapy for people with mental illnesses. There is an inadequate number of formal and high-quality evaluation studies to conclude whether or not horticultural therapy is effective for people with mental illnesses [22,23].

Several systematic reviews highlighted the limited number of quality research on HT for people with mental illness [3,24]. The structure of HT for people is diverse, and there were too many possible therapy objectives and outcome variables in HT. The recent review by Cipriani et al. [24] indicated that the key benefits of HT lies in three areas, i.e., stress and coping, mental well-being, cognitive function, self-esteem, and interpersonal relationships.

Based on these research evidence and their own pilot study [8], the New Life Psychiatric Rehabilitation Association (NLPRA) designed an eight-session standardized horticulture therapy (HT) program. The key objectives of their eight-session program for people with severe mental illness are: (1) To reduce perceived stress and anxiety and promote mental well-being; (2) to increase engagement and achievement in meaningful activities through horticulture; and (3) to increase social exchange, peer interaction, and social support, while working in a non-competitive environment. The HT groups are conducted by accredited horticultural therapists and are assisted by rehabilitation workers. The current evaluation study aimed to address the limitations of the previous studies of HT by: (1) Recruiting a sample large enough to provide adequate power in the analysis; (2) using standardized assessment instruments that align with the revised HT program; (3) providing follow-up assessments to examine if the therapy effects of HT could be maintained over a longer time frame; and (4) conducting a focus group evaluation to obtain information on participants’ experiences with horticulture therapy. The evidence from this mixed-method study is expected to contribute to the evidence-based practice of horticulture therapy for people with severe mental illness.

The objective of this study is to evaluate the effectiveness of the HT program for people with schizophrenia. We hypothesized that the HT program would impact on the outcomes variables of perceived stress and anxiety, mental well-being, and the process variables of engagement in meaningful activity, and positive mood changes during and after participation in the activity. These variables are measured at pre-test, post-test, and at a two-week follow-up. The outcome variables were measured for all participants in treatment and comparison groups, while the process variables were measured only for the participants in the treatment group.

## 2. Materials and Methods

This study uses mixed methodology to evaluate the process and outcomes of a standardized horticulture therapy group. Mixed methodology is the purposeful integration of qualitative and quantitative methods in data collection, data analysis and interpretation of evidence [25,26]. In this study, the use of mixed methodology draws on the strengths of quantitative methods in examining the outcomes of HT, while the qualitative method could explore therapy process or factors that contribute to the therapy outcomes. For the quantitative evaluation, we compared the expected outcomes between the treatment and comparison groups using standardized instruments that were conducted at pre-treatment, post-treatment, and follow-up. For the qualitative evaluation, we conducted observation ratings of the horticultural therapy group process and a focus group with participants to examine how the group processes contributed to the therapy outcomes.

### 2.1. Participants

We recruited the participants from several vocational rehabilitation service units, including sheltered workshops, supported employment or job placements. The inclusion criteria include: (1) Adults between the ages of 18 and 65; (2) a history of mental illness (participants with dual diagnosis of mental illness and mild intellectual disabilities are included); (3) regular attendance at vocational rehabilitation programs; (4) expressed an interest in horticulture and volunteered to join the HT program; and (5) attended regular follow-up appointments with psychiatrists or psychiatric services. The exclusion criteria are: (1) Co-morbid diagnoses of personality disorders, substance abuse, and organic brain disorders; (2) participation in structured horticultural therapy programs during the past six months.

A total of 82 people with mental illness agreed to join the study. They were randomly assigned to either the experimental group (*n* = 41) or the comparison group (*n* = 41). Simple randomization was conducted, using an app which generates random numbers. Participants assigned even or odd numbers are assigned to the experimental and comparison groups, respectively. This sample size, estimated using PASS 12 software [27], is adequate to achieve the power of 0.80, if we assume α is 0.05 and the effect size is medium (d = 0.5). As the standard group size for the horticulture program is 8 to 10 people, we conducted five HT groups to reach the planned sample size, assuming an attrition rate of less than 15%.

### 2.2. Horticultural Therapy Program

Based on the work of previous studies [2,28], each session of the standardized horticultural therapy program had a specific theme and therapy objectives. During the HT activities, therapists encouraged participants to share their interest in and experiences with plants and talk about their past horticulture experiences. During each horticultural therapy session, there were three phases: (1) Opening and review (five minutes); (2) horticulture therapy activity (50 minutes); and (3) closing and debriefing (20 minutes). The session titles and objectives of the eight-session horticultural activity program are listed in Table 1. The first three sessions focused on basic horticulture knowledge and skills. In Sessions 4 and 5, the therapist guided participants to use plants, fruits, and herbs as media in mindfulness and relaxation activities. In Sessions 6 and 7, the therapist conducted horticultural projects that use plants in decorations and other products, and re-visited the mindfulness techniques, and promoted reflection on the participants’ experiences. In the final session (Session 8), the therapist wrapped up the horticulture experience by harvesting vegetables that were planted in the early sessions of the group. The group cooked some of the produce, and enjoyed a meal together. Throughout the sessions, therapists guided participants to take care their own plants and observed the growth process of the plants. As the therapy session went on, the participants would take care of several types of indoor and outdoor plants during the therapy period. The therapist also guided the group to reflect their experience with horticulture, the appreciation of nature and plants, and the meaning of horticultural therapy for the participants.

### 2.3. Instruments

Demographic data, including age, gender, diagnoses, and education level, were obtained at the baseline when the participants joined the program. We used several standardized instruments to measure the outcomes of the program, administered before and after the intervention.

Stress and Anxiety. We used the Anxiety, and Stress subscales (14 items) of the 21-item Chinese version of the Depression Anxiety Stress Scale (DASS21) to monitor whether or not the HT program could reduce stress and anxiety in the participants. The DASS21 is a 21-item self-report instrument measuring current symptoms of depression, anxiety, and stress [29,30]. Respondents use a four-point scale to indicate their opinion, ranging from 0 (did not apply to me at all) to 3 (applied to me very much of most of the time). The DASS21 had adequate to good internal consistency, and Cronbach’s α for the Depression, Anxiety, and Stress subscales were 0.84, 0.77, and 0.86, respectively [31]. Taouk, Lovibond, and Laube [31] translated the DASS21 into Chinese and reported that the Chinese version of the DASS21 was sensitive to cultural and linguistic issues and could discriminate between Chinese populations with and without depression and anxiety disorders.

Mental Well-Being. We adopted the seven-item Chinese version of the Short Warwick-Edinburgh Mental Well-Being Scale (C-WEMWBS) to monitor the changes in the mental well-being of participants. Participants use a five-point scale to indicate their choices, ranging from 1 (at no time) to 5 (all the time). Validation studies of both the original English version and the Chinese version of the WEMWBS showed that it has a unidimensional factor structure, excellent reliability, and very good construct validity [32,33].

Engagement and Meaningfulness. The Engagement in Meaningful Activities Survey (EMAS) is a 12-item questionnaire that assesses the perception of how far the activity that one has participated is personally meaningful [34]. It is used to capture data about the participants’ sense of engagement, accomplishment, and meaningfulness regarding HT. Respondents use a four-point rating scale to indicate their agreement with test items, ranging from 1 (rarely) to 4 (always). The EMAS has a unidimensional factor structure and very good reliability, and its scores correlated in expected directions with the concurrent measures of well-being and life satisfaction [35]. After getting permission from the author, we conducted forward and backward translations of the instrument and prepared it for use in this study. As it is necessary to ask participants to refer to a specific activity when answering questions related to its meaning, we could only conduct the EMAS with participants of the HT (treatment) group.

Engagement and Affect during HT Sessions. We hypothesized that HT could promote positive emotions and participants’ engagement in activities. Using the observation and recording method developed by Gigliotti and Jarrott [36], trained assessors rated the engagement and the predominant affect of each participant using a behavioral sampling schedule. Trained observers rated the engagement and affect of participants during five-min intervals in the group. To rate the participants’ engagement, the assessor assigned one of four behavior codes reflecting their engagement: (1) Social (S); (2) horticultural therapy (HT); (3) productive (P); and (4) nothing (N). For the rating of affect, the assessor rated participants’ facial expressions on a six-point scale ranging from −5 (extremely negative), to −3 (moderately negative), −1 (mildly negative), +1 (mildly positive), +3 (moderately positive), and +5 (very positive). We conducted these observational ratings in the 2nd and last sessions of the HT program, so that we could compare the affect and engagement of subjects over the group process.

Social Exchange and Support Measure (SESM). We adopted the self-administered questionnaire developed by Brown, Tang, and Hollmanto [37] to measure how participants may benefit from the social exchange during group participation. Respondents use a 7-point scale ranging from 0 (0 times) to 7 (10 times or more) to rate the frequency of social exchanges. The SESM measures six types of social exchanges in therapy groups, including experiential knowledge provided, emotional support, experiential information, humor, unwanted behavior, and exchanges outside meetings. The measure demonstrates good model fit with theoretical constructs and shows evidence in support of it convergent and discriminant validity. We compared participants’ ratings made in the first and final sessions of the HT program.

### 2.4. Procedures

We obtained ethical approval to conduct this study from The Research Committee, Department of Rehabilitation Sciences, Hong Kong Polytechnic University (Reference Number HSEARS20160618001, dated 29 June 2016). All subjects were recruited from the rehabilitation service and centers under the NLPRA. During a briefing session, research staff explained to potential subjects the purpose and procedures of the study and how they could contribute to the study process. Subjects who were willing to participate signed the study’s consent form.

**Figure 1:**
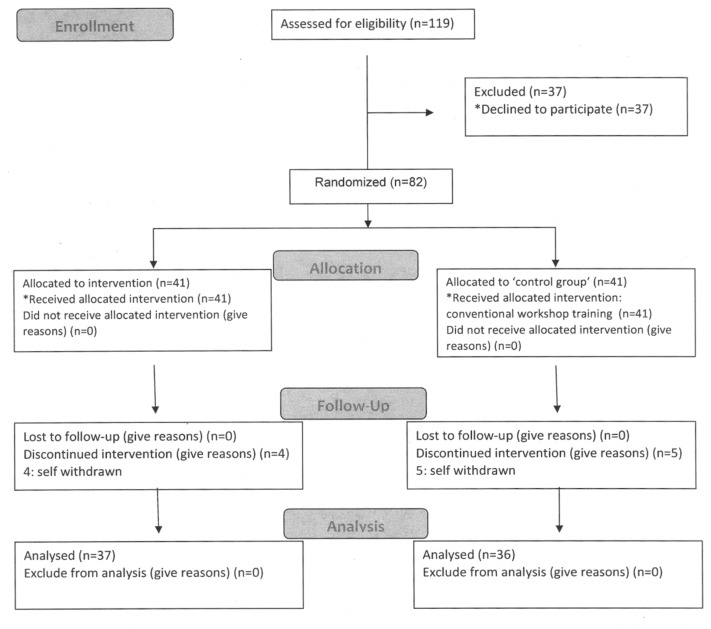
Consort Flow Diagram for the quantitative study of horticultural therapy program.

For the quantitative study, the subjects were recruited from vocational rehabilitation programs. We approached a total of 119 persons with mental illness, and 37 declined to join the study (Figure 1). Eighty-two participants were randomly assigned to the treatment and comparison groups, and both continued to attend their usual vocational rehabilitation program. The treatment group subjects attended the horticulture therapy program once a week for eight weeks. Participants in the comparison group continued with their usual training in sheltered workshops or work placements during the study period. The usual training activities include work-related tasks (craft or manufacturing work), simulated work training, and coaching is provided at job placement sites.

Two self-completed questionnaires on outcomes are conducted. Stress and anxiety were assessed using the DASS21 subscales before the start of HT (pre-test), when the last session is completed (post-test), and at the two-week follow-up. The mental well-being measure (C-SWEMWBS) was conducted only at pre-test and follow-up, as changes in mental well-being are less immediate when compared with changes in anxiety and stress. The process measures on affect changes (measured by Affect & Engagement Checklist), engagement in meaning activity (EMAS), and social exchange (measured by the Social Exchange and Support Measure) were completed at pre-test and post-test. The rating of these measures was linked to the observation of subject performance in the HT group, and it is not meaningful to conduct them during follow-up. Blinded assessors distributed the questionnaires and collected the completed questionnaires from the participants. The observational assessment of affect changes was completed during the first and final two sessions of the HT program by trained research assistants.

### 2.5. Focus Group Evaluation

We invited eight horticultural therapy participants to join the focus group. Five are female, and three are male, and their mean age is 47 (SD = 10.7). The objectives of the focus group were to explore the experiences of the clients in the program, how they perceived the impact and outcomes of horticulture therapy, and what the structure and process factors that promote the satisfaction and outcomes are. A semi-structured interview guide, with suggested questions, was used to explore the following areas of the focus group: (1) Motivation to join horticultural therapy; (2) group structure and process; (3) factors linked to participation in and satisfaction with horticultural therapy; (4) impact and outcome of the program; (5) and social interactions in and dynamics of the HT group. The researcher, a qualified counsellor, conducted the focus group and a research assistant assisted in the arrangement and recording of the session. The researcher is not involved in the implementation of horticultural therapy, and the two horticultural therapists who conducted the therapy groups did not take part in the focus group.

### 2.6. Quantitative Data Analysis

The data collected using DASS, EMAS, and C-SWEMWBS were analyzed using repeated measures General Linear Model (GLM) to examine group differences while accounting for the variance, due to changes over time. An ANCOVA would be used instead of a repeated measures ANOVA if we found that there were significant differences in the baseline scores of the measures. For SESM (social exchange measures), we used a paired t-test to compare the pre- and post-scores of the treatment group. The qualitative data collected for the group evaluation were analyzed using content analysis. The interviews were transcribed and coded, then key themes regarding the participants’ experiences in HT were identified.

### 2.7. Qualitative Data Analysis

We used the framework developed by Rabiee [38] as a guide in data analysis. The voice recording of the focus group is transcribed, and we conducted a content analysis to identify the key themes about the perceived outcomes of HT, and the factors and therapeutic process affecting the outcomes. The transcribed text of the interview is coded, and coded text with similar content and meaning are summarized. We grouped and checked if the contents addressed the guided questions. In the interpretation of data, we considered the following attributes of the data (comments): (1) Frequencies and extensiveness; (2) intensity; (3) internal consistency; (4) the big ideas. The key meaning, themes, and big ideas obtained from qualitative data analysis are triangulated with the results of quantitative analysis, whenever it is relevant.

## 3. Results

### 3.1. Profile of Participants

There were slightly more female (*n* = 45, 54.9%) than male (*n* = 37, 45.1%) participants, and their average age was 50.3 years old (SD = 9.6). Most of the participants had received a junior secondary education (35.4%) or a senior secondary education and above (40.2%). About three-quarters of the participants (76.8%) had received a diagnosis of schizophrenia. The average onset of illness was 25.0 years (SD = 10.7). All participants attended vocational rehabilitation programs or job placements during the day. The participants lived with their families (36.6%), in hostels or halfway houses (37.8%), or alone (25.6%). We did not find significant differences in the demographic profiles of participants in the experimental treatment group and the comparison group (Table 2), except in regard to their living conditions (χ^2^ = 8.78, *p* < 0.01). More participants in the treatment group lived in hostels or halfway houses than in the comparison group.

### 3.2. Quantitative Evaluation

There were significant differences in the outcome variable of mental well-being, as measured by the C-SWEMWBS (*F* = 6.5, *p* = 0.01), as well as in the process variables of engagement in meaningful activity measured by the engagement scale of the AEC (*F* = 12.45, *p* = 0.00), and the EMAS (*F* = 3.53, *p* = 0.06) between groups. The treatment group participants had significantly higher levels of mental well-being, showed more engagement in horticultural activities, and found the activities more meaningful than did the comparison group (Table 3).

There were no significant differences in outcomes variables of anxiety and stress (measured by DASS), between the treatment and comparison groups in regard to affect during activity (AEC affect scale), and social exchange (SESM). The participants had very low anxiety and stress scores in both DASS subscales, which remained very similar over time. The AEC affect scores remained neutral over time.

Overall, the study results indicated that HT promoted mental well-being, but did not significantly reduce of stress and anxiety of participants. Horticultural therapy could significantly promote the engagement of participants in meaningful activities, but we could not observe significant changes in affect among the subjects during HT sessions. There was also no significant increase in social exchange among the subjects.

### 3.3. Focus Group Evaluation

None of the participants had joined any horticultural activities or forms of horticultural therapy before the current study and did not have any prior concrete expectations. We identified five themes from the conversations in the focus group interview (Table 4). First, many participants expressed their satisfaction with end products of horticultural activities, and it appeared that the end product motivates them to participate in HT. Many participants mentioned it is good that they could have edible produce from horticulture. Others recalled the pleasing smell of aromatic herbs, and they could use them to make drinks or tea, or expel insects. Many also recalled the handcraft work they had done, such as making dry flowers, which they could send to friends and family as gifts. 

Second, horticultural activities have stimulated the knowledge and skill acquisition of participants. Many mentioned that they learn a lot about plants and wish to join more advanced classes if available. They appreciated the knowledge and skills they have acquired, such as how to mix soil, how to use fertilizers, how to water plants, or the practical uses of some plants. 

Third, about half of the participants said they were amazed at the vitality and resilience of plants. Some participants gave examples like outdoor plants could grow better and stronger after severe storms, and it is pleasurable to take care of plants as they grow up. Other participants mentioned that the group enabled them to appreciate nature and plants, and many became motivated to learn more skills regarding how to take care of plants and nature.

Fourth, the horticultural activities stimulated their interest in horticulture and expanded their horizons in regard to understanding and taking care of plants. One participant mentioned that he wanted to pursue a career in horticulture, while most of the other participants stated they would like to further engage in horticultural activities as a hobby. One said he would like to rent a small piece of land on a farm, so that he could enjoy small-scale farming and horticulture in the future.

Last, the participants mentioned some psychological, social, and spiritual benefits of horticultural activities and therapy. Regarding the psychological benefits, participants said that horticulture activities had helped them to develop patience and peacefulness, relieve stress, and let them practice their thinking skills. Many said they shared group photos, logbooks, and end products with peers and family members. Regarding the spiritual benefits, they said they had learned to appreciate the miracle of life. Some said they had developed an ability to focus and put their energy in activities. 

Overall, most participants had positive views of the program. It stimulated their interest in horticulture and motivation to learn more about it. Many mentioned that they had started to appreciate nature and were amazed by the vitality of plants. It was evident that most participants engaged well in the HT group, enjoyed the therapy process, felt satisfied with their participation, and found horticultural activities meaningful.

## 4. Discussion

### 4.1. Outcomes and Processes of Horticultural Therapy

We combined the quantitative and qualitative results and interpreted them to examine the outcomes and process of HT, and there are several important observations. First, the quantitative results indicated that horticultural therapy made a positive impact on mental well-being. This is supported by a significant change in the mental well-being scores of the treatment group, when compared with the comparison group. The change in mental well-being could be explained by the theme of psychosocial and health benefits described by participants of the focus group. Participants said that horticultural activities help them stay calm, develop patience, relieve stress, stay focus on tasks, and use their brains more. They also have more share their experience with friends and family, which all could contribute to their psychosocial well-being. Many previous studies of HT used physical or general well-being as an outcome indicator [39,40,41], but few use mental well-being scales. Researchers could consider using mental well-being scales for studies of HT in mental health.

Second, the quantitative results from the questionnaire showed that subjects did not have a significant reduction in stress or anxiety, and this is consistent with the observational ratings of affect. This is, however, inconsistent with our pilot study [8] and many other studies of HT [39,42], as well as the perceptions of focus group participants who said that they experienced relief of stress and anxiety. The insignificant change in stress and anxiety could be explained by a flooring effect of the stress and anxiety scores at pre-test. It means that many subjects had very low stress and anxiety, and it could be hard for these scores to further decrease after HT. The other alternative explanation is that many people with schizophrenia have difficulties in emotional expression [43,44], and it could be challenging to detect such changes from observational ratings.

Third, it is clear from the quantitative results (questionnaires and observational ratings) that HT could raise the engagement and participation of people with mental illness. This is consistent with the results of several studies that use engagement measures in studying HT [36]. Engagement measures reflected participants experience meaning and success in participation, and could be routinely used in the evaluation of motivation for participation in people with more severe disabilities. The qualitative study results highlighted several possible reasons supporting the engagement in HT, including the enjoyment of end products of horticultural activities, the acquisition of knowledge and skills in horticulture, or the realization about vitality and resilience of plants through the activities.

Last, we noted that the HT group had no significant impact on social exchanges among subjects. During the focus group, participant seldom mentioned their social experience in the group, or that they expect to make friends or learn to communicate better in the HT group. The main point they highlighted the successful social experience is that they find it pleasurable to share photos and end products of horticultural activities with friends and family. As reflected by the therapists, it is challenging to stimulate sharing, reflection, and discussion after the activities, due to lack of time. They also notice that many subjects tended to share more about the concrete aspects (e.g., end products) of the group, rather than a reflection of feelings or meaning in group participation. Indeed, there are only a few studies that investigated if HT could promote increase group cohesiveness or social interaction [17,45]. Further study on this therapeutic benefit of HT is needed.

### 4.2. Contributions and Limitations of the Study

The current study is an important addition to the literature evaluating horticultural therapy, as it addresses a number of methodological deficiencies in the current literature, It used random assignment, included a comparison group, recruited a sample that provides adequate power for analysis, and both the HT program and outcome measures were standardized. It was also one of the few evaluation studies that used a mixed methodology to evaluate HT, which could provide more comprehensive, internally consistent, and valid findings through different methodological approaches, thereby providing richer information to address the research questions [25,26].

Nevertheless, the design of this study still had several limitations. First, the participants in both the treatment and comparison groups attended a vocational rehabilitation program during the day, and the treatment group participants were released once a week from their regular program to attend the HT. The comparison of the treatment group with a treatment-as-usual group could lead to a smaller effect size, as the usual program could also contribute to positive changes in the outcome measures. Second, there are changes that may not be fully captured by the design and measures of this study. Future studies could, for example, monitor changes in participants’ interest in plants, and their horticultural knowledge and skills. Third, we faced challenges in examining participants’ changes in satisfaction and affect associated with HT. While many said they were very satisfied with the HT group during the focus group, we did not observe significant changes in the emotional expression of participants over the group sessions. Many people with schizophrenia, express emotions less and many find it hard to share their emotions or their own experience [44,45]. This could be why we could only capture a significant increase in the participants’ engagement and participation in group activities. The focus group participants also tended to share their experiences related to the end products and their horticultural knowledge and skills, rather than reflections on their experiences and meaning found in the sessions. Last, most of the participants had a diagnosis of schizophrenia, the study results would mainly be applicable to people with this diagnosis. In fact, schizophrenia is the most common diagnosis of clients attending vocational rehabilitation services in Hong Kong.

## 5. Conclusions

The study results indicate that the HT program was effective in increasing the engagement, mental well-being, and sense of meaningfulness and accomplishment of the participants. Although many participants stated that HT helped them to reduce stress and anxiety during the focus group, positive changes in affect were not fully observed during the group process and were not captured by standardized measures of stress and anxiety. The participants also did not report increases in social exchange, support, or cohesiveness over the course of the HT sessions. To further enhance the impact of the HT program, therapists could consider focusing more on the sensory experience of HT. To promote social exchange and support, therapists need to allow more time for debriefing and sharing. The sharing process could be facilitated by an appreciation of the end products of the sessions, and by referring to artefacts, photos, and logbooks created during the sessions. Researchers could consider measuring the changes in horticultural knowledge and skills, or further assessing changes in participants’ interest in and motivation regarding horticulture or daily activities. Qualitative methods, such as focus group interviews or reflective journals, could be used to capture the spiritual and psychological aspects of HT.

## Figures and Tables

**Table 1:** Session titles and objectives of the horticulture therapy (HT) program.

Session Title	Session Objectives
Growth with Hope: Transplant Sprouting Seedling Activity	To learn and practice transplantation. To share the objectives and expectations of the HT sessions.
The Lifecycle of Plants: Propagation by Cutting	To learn and practice propagation skills. To reflect on, appreciate nature, and share thoughts about the vitality of plants.
The Lifecycle of Plants (II): Propagation by Division	To learn and practice propagation skills. To reflect on, appreciate nature, and share thoughts about the vitality of plants.
Mindful Eating: Tasting Fruits	To experience mindfulness techniques during the activities. To share thoughts about using this experience in mindful eating, and its potential use as a relaxation activity.
Live Aroma: Introduction to Herbs	To introduce herbal plants to the users. To learn and practice a diaphragmatic breathing exercise.To share thoughts about the experience of using relaxation methods.
Self-Designed Pot Garden: Combination of Plants	To learn to design a pot garden using various plants and decorations. To share thoughts about the feeling of appreciation.
Natural Aroma: Herbal Bag Production	To experience the aromas of various kinds of dried natural materials. To revise the diaphragmatic breathing exercise.
Harvest Time: Vegetable Harvesting	To practice vegetable harvesting and cooking. To share the symbolic meaning of harvesting.To share overall feedback about the HT sessions.
If the therapy setting does not have an outdoor environment for vegetable growth, Sessions 1 and 8 will be changed to sessions entitled “Colorful Nature” and “Colorful World”, described below.	
Colorful Nature: Leaf Rubbing	To experience the natural colors of plants through a leaf rubbing exercise. To learn about the characteristics of plants.
8. Colorful World: Pressed Flower Cards	To learn skills using pressed flowers. To share the symbolic meaning of pressed flowers. To share overall feedback about the HT sessions.

**Table 2:** Comparison of the demographic profiles of the treatment and comparison groups.

Variables	Group				χ^2^	*p*
	Treatment		Comparison			
	n	%	*n*	%		
**Categorical Variables**						
Gender						
Male	20	54.1%	17	45.9%	0.44	0.51
Female	21	46.7%	24	53.3%		
Living Condition						
Live with family	11	36.7%	19	63.3%	8.78	0.01
Hostel	22	71.0%	9	29.0%		
Live alone	8	38.1%	13	61.9%		
Diagnosis						
Schizophrenia	30	47.6%	33	52.4%	0.62	0.43
Other psychiatric illness	11	57.9%	8	42.1%		
Education						
No formal education	2	100.0%	0	0.0%	2.34	0.67
Special education	1	50.0%	1	50.0%		
Primary	8	50.0%	8	50.0%		
Junior secondary	13	44.8%	16	55.2%		
Senior secondary or above	17	51.5%	16	48.5%		
**Interval Variables**	M	SD	M	SD	t	p
Age	50.8	10.5	49.7	8.7	0.51	0.61
Years from onset	25.6	11.6	24.4	9.9	0.50	0.62

**Table 3:** Comparison of the outcomes of the treatment and control groups.

Measures	T1 Pre-Test	T2 Post-Test	T3 Follow Up	Test Statistic	*p*
**DASS**					
Anxiety subscale					
Treatment	0.59 (0.55)	0.64 (0.54)	0.72 (0.59)	1.14 ^a^	0.29
Comparison	0.71 (0.47)	0.79 (0.72)	0.84 (0.68)		
Stress subscale					
Treatment	0.73 (0.66)	0.71 (0.66)	0.87 (0.68)	0.85	0.36
Comparison	0.85 (0.48)	0.84 (0.65)	0.96 (0.66)		
**C-SWEMWBS**					
Treatment	3.10 (0.66)	---	3.30 (0.73)	6.50 ^b^	0.01
Comparison	3.18 (0.71)	---	2.95 (0.70)		
**EMAS**					
Treatment	2.72 (0.62)	2.74 (0.69)	---	3.53 ^b^	0.06
Comparison	2.58 (0.69)	2.40 (0.74)	---		
**AEC**					
Affect					
Treatment	0.035 (1.15)	0.40 (1.07)	---	1.05 ^b^	0.31
Comparison	−0.29 (0.72)	0.22 (1.00)	---		
Engagement (%)					
Treatment	97.06 (5.67)	96.16 (7.06)	---	12.45 ^b^	0.001
Comparison	83.33 (21.91)	81.00 (18.14)	---		
**SESM**					
Experiential knowledge provided	2.98 (1.73)	2.70 (1.72)	---	1.12 ^c^	0.27
Emotional support provided	3.63 (1.70)	3.48 (1.63)	---	0.70 ^c^	0.49
Experiential knowledge received	3.15 (1.63)	3.29 (1.48)	---	0.53 ^c^	0.60
Emotional support received	3.61 (1.70)	3.95 (1.59)	---	1.53 ^c^	0.13
Humor exchanged	2.32 (1.64)	2.28 (1.20)	---	0.12 ^c^	0.90
Unwanted behavior received	1.52 (1.13)	1.54 (.94)	---	0.10 ^c^	0.92

Note. DASS = Depression Anxiety Stress Scale; C-SWEMWBS = Chinese Version of the Short Warwick-Edinburgh Mental Well-Being Scale; AEC = Affect and Engagement Checklist; EMAS = Engagement in Meaningful Activities Survey; SESM = Social Exchange and Support Measure. ^a^ Repeated measures ANOVA was used. F test of Time *x* Group Interaction term is reported. ^b^ ANCOVA was used, as there were significant differences in the baseline scores between the treatment and control groups. *F* test of Time *x* Group Interaction term is reported. ^c^ A paired t-test was used for the comparison of the pre- and post-test scores of the treatment group. We did not conduct this questionnaire with the comparison group.

**Table 4:** Themes identified from analysis of focus group interview, with quotations of participants.

Theme	Quotations of Participants
1. Enjoying End Products of Horticulture	I can grow and eat strawberries, if I am able to grow it.
	I learn about the smell of different herbs
	I love herbal tea now.
	I learn to make herbal bags, they are beautiful, smells good, and could expel the insects.
	It is important to grow something that I can eat.
	I like to have produce from horticulture.
	Herbal could be used in seasoning
	I learn to press and preserve flowers and grass, for making small gifts.
2. Knowledge & Skill acquisition	I never know there are plants like this … such as “mini coconuts”.
	I learn to grow tangerine from seeds.
	I want to learn more, eight sessions is not enough.
	I want to learn more about how to use fertilizers, and how to mix soils for different plants.
	There is so much to learn in horticulture.
3. Vitality and resilience of plants.	I remember our horticulture class was suspended due to typhoon that day, but the plants grow even bigger and stronger after the severe storm.
	It is pleasurable to see the shoots growing into plants.
	Plants are amazing.
	Some plants and flowers will close at night, but re-open in the morning.
	I did not realize that plants are so resilient.
4. Serious leisure and potential career development	Horticulture could be a kind of play, but it also could be work.
	After learning horticulture, I have more and more plants at home, such as tomatoes, cactus, etc.
	I see people working in gardens or parks, they must know about horticulture. I am interested in this kind of work.
	It may be good for me to open a flora store. Maybe we could run a stall at the annual Flower Market.
5. Psychosocial and health benefits	I can focus, put all my energy into it.
	Horticulture helps me to stay calm, practice patience.
	Gives me lots of good memories
	Help me relieve stress
	I become more agile, I use my brain more.
	I took photos of my plants and share with friends and family.
